# Active Inference with Dynamic Planning and Information Gain in Continuous Space by Inferring Low-Dimensional Latent States

**DOI:** 10.3390/e27080846

**Published:** 2025-08-09

**Authors:** Takazumi Matsumoto, Kentaro Fujii, Shingo Murata, Jun Tani

**Affiliations:** 1 Cognitive Neurorobotics Research Unit, Okinawa Institute of Science and Technology, Okinawa 904-0495, Japan; takazumi.matsumoto@oist.jp (T.M.); jun.tani@oist.jp (J.T.); 2 Department of Electronics and Electrical Engineering, Keio University, Kanagawa 223-8522, Japan; oakwood.n14.4sp@keio.jp

**Keywords:** free-energy principle, active inference, information gain, goal-directed action planning, curiosity-driven exploration

## Abstract

Active inference offers a unified framework in which agents can exhibit both goal-directed and epistemic behaviors. However, implementing policy search in high-dimensional continuous action spaces presents challenges in terms of scalability and stability. Our previously proposed model, T-GLean, addressed this issue by enabling efficient goal-directed planning through low-dimensional latent space search, further reduced by conditioning on prior habituated behavior. However, the lack of an epistemic term in minimizing expected free energy limited the agent’s ability to engage in information-seeking behavior that can be critical for attaining preferred outcomes. In this study, we present EFE-GLean, an extended version of T-GLean that overcomes this limitation by integrating epistemic value into the planning process. EFE-GLean generates goal-directed policies by inferring low-dimensional future posterior trajectories while maximizing expected information gain. Simulation experiments using an extended T-maze task—implemented in both discrete and continuous domains—demonstrate that the agent can successfully achieve its goals by exploiting hidden environmental information. Furthermore, we show that the agent is capable of adapting to abrupt environmental changes by dynamically revising plans through simultaneous minimization of past variational free energy and future expected free energy. Finally, analytical evaluations detail the underlying mechanisms and computational properties of the model.

## 1. Introduction

Active inference (AIF), rooted in the free-energy principle (FEP) [1], is a theory of cognition that provides a unified framework for agent perception and action [2,3]. An active inference agent selects actions that minimize expected free energy (EFE); when the agent has a prior preferred outcome, searching for the lowest EFE naturally selects actions that change the world state toward the preferred outcome. In most studies, AIF has been applied in discrete action spaces as a Markov decision process; however, in this study we focus on a partially observable continuous action space.

Our previous work proposed a novel approach to active inference for goal-directed planning [4,5], wherein goal-directed plan search in a continuous action space can be performed efficiently by inferring low-dimensional latent variables. This contrasts with recent related studies that formulate AIF in a continuous space with a policy search scheme [6,7].

The internal predictive model learns to predict proprioceptive–exteroceptive sequences by which habituated behaviors are generated. Note that the predictive model does not predict motor sequences directly but predicts proprioceptive sequences for generating movement trajectories by using a simple inverse model. Such habituated behaviors are internally represented with prior probability distributions over latent variables in a generative model. Following inference, a goal-directed action plan can then be generated using the predictive posterior distribution, under the constraint of prior preferences or goals.

This makes the search space significantly smaller, as habituation naturally constrains the possible behavior space, i.e., goal-directed planning avoids searching a large space of unfamiliar trajectories. However, a disadvantage of this approach is that the hidden states are under-explored, whereas typical AIF approaches employ an epistemic term (information gain) to generate curiosity-driven exploration [8,9].

In this study we propose EFE-GLean, an extension to our previous T-GLean model that enables the agent to explore for hidden information in the environment, allowing it to reliably achieve goals by leveraging information gain while still searching for plans in the lower-dimensional latent space. Details of our proposed model are given in Section 3.

Our proposed model is evaluated in Section 4 in a series of simulation experiments within a T-maze with colored floors, following [3], under both discrete and continuous action domains. Our experiments demonstrate that our agent is able to (i) generate efficient goal-directed behavior by exploring hidden states in the environment, and (ii) rapidly adapt to sudden environmental changes by error regression.

## 2. Related Work

The free-energy principle proposed the concept of variational free energy (VFE, also known as evidence free energy $F_{e}$) as an objective function for the brain, where neural dynamics perform variational inference balancing the accuracy of its predictions of observations *x* (evidence) and deviation between the posterior *q* and prior *p* beliefs over hidden states *z* [1]. Active inference incorporates the concept of action as a means of reducing VFE [2,10]. Free-energy minimization has been explored extensively in the literature, and we describe our own approach, based on [5], in Section 3.

(1) $$\begin{array}{cc} F_{e}\left(x\right) & =E_{q}\left[\ln q(z)-\ln p(x,z)\right]\\ \; & =\underset{\mathrm{Divergence}}{\underset{︸}{D_{KL}\left[q\left(z\right)||p\left(z|x\right)\right]}}-\underset{\mathrm{Log evidence}}{\underset{︸}{E_{q}\left[\ln p(x)\right]}}\; \end{array}$$

Expected free energy (EFE) extends the temporal domain into the future [2,11] by estimating the free energy of the agent after executing the actions in a given policy π. A key challenge is thus how can we find an optimal policy (or plan) that has the lowest EFE.

EFE contains two contrasting objectives: goal-directed planning and curiosity-driven exploration. For the former, the agent can generate future actions and predicted observations, and if the agent has a preferred observation, then by maximizing the extrinsic value it is possible to search for plans that have actions that result in that preferred observation [12,13]. Epistemic value (also referred to as information gain) quantifies the anticipated uncertainty in a given future state. By maximizing the epistemic value, the agent seeks states of high uncertainty, resulting in curiosity-driven exploration behavior [8,9].

(2) $$\begin{array}{cc} G(\pi ) & =E_{q(x,z|\pi )}\left[\ln q(z|x,\pi )-\ln p(z,x)\right]\\ \; & =-\underset{\mathrm{Epistemic value}}{\underset{︸}{E_{q(x|\pi )}\left[D_{KL}\left[q\left(z|x,\pi \right)||p\left(z\right)\right]\right]}}\;-\underset{\mathrm{Extrinsic value}}{\underset{︸}{E_{q(x|\pi )}\left[\ln p(x|C)\right]}}\; \end{array}$$

While AIF provides a powerful framework for exploration–exploitation agents, in many earlier studies, agents have been limited to a discrete action space. This is sufficient for agents in simple environments such as a grid world [2] or any environment that can be discretized into several states [3]. While our focus is on continuous state spaces, we will also explore a discrete action space based on the T-maze described in [3] in Section 4 for the purpose of examining the essential characteristics of AIF in simpler settings.

When scaling up to a continuous domain, enumeration of all possible policies becomes impractical [9]. Many proposals employ deep neural networks for the world model and/or policy generation, which can be trained by reinforcement learning (RL) [6,14]. Such approaches typically also employ a policy search (roll-out) approach that samples many policies to search for a plan with the lowest EFE [9]. While this approach is synergistic with traditional RL implementations, the policy generation and roll-out procedure is computationally expensive, with the number of samples to search for the lowest EFE typically on the order of 100 [15].

Several approaches avoid policy roll-out completely by considering the current action as a variable to be optimized by gradient descent [16,17,18] using free-energy minimization. While these approaches are fast enough to be employed as robot controllers (updating at several kHz), they lack the ability for long-horizon planning since they only consider variational free energy in order to predict the next step. This limits their ability to plan around sudden obstacles and consider multi-step actions to reach the goal. We compare the performance of agents performing only variational free-energy minimization in the absence of sample-based evaluation of EFE in Section 4.

A prior work [15] utilized a world model [19,20] and a policy suggester that generates candidate policies from a list of all possible policies. The key contribution of that work was the incorporation of a “preference precision” hyperparameter that allows the experimenter to switch the agent’s behavior between an exploratory and goal-directed mode. This was demonstrated on a physical arm robot attempting to move a colored ball that is obscured by a lid. By adjusting preference precision to a small value, the robot preferred to maximize epistemic value by moving the lid, while a large preference precision value caused a preference for actions that led to matching the preferred observation. In the current work, we leverage a predictive-coding-inspired variational recurrent neural network (PV-RNN) as both a world model and policy generator, and directly estimate the preference precision from future uncertainty.

Our prior work on goal-directed action plan generation, T-GLean [4,5], focused on efficient search in lower-dimensional latent space by maximizing extrinsic value, provided that sufficient human tutoring for appropriate behaviors is offered. The key advantage of this approach is a significantly smaller search space, with gradient descent in the latent space being sufficient to find an optimal plan. However, omitting the epistemic value from expected free energy removes the intrinsic motivation for self-exploring hidden information, which may be critical for generating optimal action plans.

In this work, we propose EFE-GLean, an extension to T-GLean that incorporates epistemic value and preference precision. Our new approach allows our agent to maximize information gain while maintaining action plan generation in lower-dimensional latent space instead of higher-dimensional policy space. This feature can be an advantage which becomes especially salient for real-world robots that have many degrees of freedom, rendering the action space much larger than the latent space.

## 3. Methodology

### 3.1. Model Architecture

**Figure 1:**
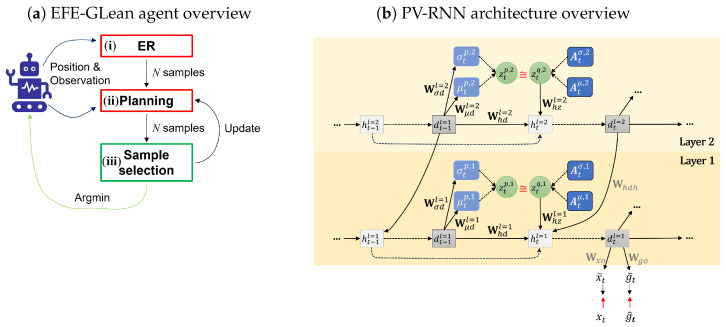
(**a**) An overview of our EFE-GLean agent with three main components: (i) error regression (ER), (ii) plan generation, and (iii) sample selection. (i) and (ii) are implemented using a 2 layer PV-RNN, as shown in (**b**) for a single time step *t*. The W and A variables are learned during training. The computation of the stochastic latent variable *z* and deterministic variable *d* are given in this section. Note that to reduce complexity, in subsequent discussion we consider only a single-layer PV-RNN, without the fully connected output.

Our newly proposed model, EFE-GLean, extends T-GLean [4] and also employs a PV-RNN architecture [21]. Figure 1 shows an overview of our agent using EFE-GLean with the three main components that we describe in this section.

(i) Error regression (ER) contains both the operation to update states in the past window and predict future states using the past approximate posterior, while (ii) plan generation infers future posterior distributions. (iii) Sample selection calculates EFE and selects the plan to execute on the agent, after which the approximate posterior of (ii) is updated as the average of all candidate plans.

As previously noted, the error regression and plan generation components are implemented using a PV-RNN. A schematic of the PV-RNN is shown in Figure 1b. In this section we describe the most pertinent parts of the model, followed by our proposed extension for exploratory behavior. Note that although the employed PV-RNN has two layers, we describe the methodology using a single layer for simplicity. As in our previous work [4], the framework receives the observation $x_{t}$ and the goal $\hat{g}$ at each time step *t*. Here, the observation $x_{t}$ comprises both proprioceptive and exteroceptive observations, but we refer to them collectively unless stated otherwise. In this work, the goal $\hat{g}$ depends only on the environment, not on time step *t*. The framework predicts the observation ${\bar{x}}_{t}$ and the goal ${\bar{g}}_{t}$ on the basis of the deterministic latent state $d_{t}$. The outputs ${\bar{x}}_{t}$ and ${\bar{g}}_{t}$ are computed using a linear projection; however, for brevity we will omit discussion of the output layer. The deterministic latent state $d_{t}$ is inferred from the stochastic latent state $z_{t}$, which is computed using the reparameterization trick [22]. The formulation for $d_{t}$ is given in Equation (3). $W_{hd}$ and $W_{hz}$ are learned connectivity weight matrices, with the bias term omitted for brevity. Similarly, we also omit further discussion of the intermediate hidden state $h_{t}$ and refer directly to $d_{t}$.

(3) $$\begin{array}{cc} z_{t} & =\mu_{t}^{\cdot }+\sigma_{t}^{\cdot }\epsilon ,\;\epsilon ∼N\left(0,1\right),\\ h_{t} & =\left(1-\frac{1}{\tau }\right)h_{t-1}+\frac{1}{\tau }\left(W_{hd}d_{t-1}+W_{hz}z_{t}\right),\\ d_{t} & =\tanh(h_{t}). \end{array}$$

As shown in Equation (4), the prior distribution $p(z_{t}|d_{t-1})$ over the stochastic latent state $z_{t}$ at time step *t* is conditioned on the deterministic latent state $d_{t-1}$ from the previous step. Again, $W_{\mu d}$ and $W_{\sigma d}$ are learned connectivity weight matrices.

(4) $$\begin{array}{cc} \mu_{t}^{p} & =\tan h(W_{\mu d}d_{t-1}),\\ \sigma_{t}^{p} & =\exp(W_{\sigma d}d_{t-1}). \end{array}$$

In contrast, the approximate posterior $q(z_{t}|x_{t},\hat{g})$ is conditioned on the corresponding observation $x_{t}$ and the goal $\hat{g}$. More precisely, as shown in Equation (5), the approximate posterior depends on a learnable variable, the adaptive vector $A_{t}$; by updating $A_{t}$ according to $x_{t}$ and $\hat{g}$, the posterior distribution is effectively conditioned.

(5) $$\begin{array}{cc} \mu_{t}^{q} & =\tan h(A_{t}^{\mu }),\\ \sigma_{t}^{q} & =\exp(A_{t}^{\sigma }). \end{array}$$

### 3.2. Learning

During learning, we minimize the following evidence free energy in Equation (6) across the entire sequence length by iteratively updating the approximate posterior and the PV-RNN learnable parameters at each time step *t* for the whole training sequence:

(6) $$\begin{array}{cc} F_{e}\left(x,\hat{g},z\right)=\sum_{t} & (w\cdot D_{KL}\left[q\left(z_{t}|x_{t:T},{\hat{g}}_{t:T}\right)∥p\left(z_{t}|d_{t-1}\right)\right]\\ & -E_{q(z_{t}|x_{t:T},{\hat{g}}_{t:T})}\left[\log p(x_{t},{\hat{g}}_{t}|d_{t})\right]). \end{array}$$

Here, *w* is a deterministic hyperparameter, called the meta-prior, that controls the degree of regularization. The meta-prior regulates the way of adapting the prior distribution of the random latent variables at every time step in the case of learning with minimization of evidence free energy, as shown in [21]. In that previous work, it was shown that learning tended to overfit to training data with a high *w* value and vice-versa with a low *w* value. Therefore, adequate setting of *w* is necessary to achieve successful learning of PV-RNN.

The first term of the evidence free energy is the Kullback–Leibler (KL) divergence between the approximate posterior and the prior over the stochastic latent states *z*. The second term is computed as the squared error—i.e., the difference—between the generated output and the training target. Once these terms are evaluated, gradients of the evidence free energy are propagated backward through time (BPTT) [23] from the end of the training sequence to the first time step. After learning, all model parameters are frozen except for the adaptive vectors. By optimizing these adaptive vectors, the agent can adapt to a new environment and generate action plans. Effectively, this is a form of deep active inference in which the recurrent neural network supports inference through the optimization of adaptive vectors. Unlike standard amortized inference—which learns a direct mapping from observations to approximate posteriors—posterior beliefs here are determined solely by the optimized adaptive vectors.

### 3.3. Exploratory and Goal-Directed Action Plan Generation

To realize both exploratory and goal-directed behavior while taking current and past observations into account, we compute free energy in two separate windows—past and future. We first describe the free-energy formulation used in each window. For the past window, we minimize the evidence free energy in the same manner as during the learning phase. Conversely, for the future window, we minimize the EFE, defined below, to generate an action plan:

(7) $$\begin{array}{cc} G\left(x,\hat{g},z\right)=\sum_{t=t_{c}}^{t_{c}+\tau_{f}} & (-E_{p\left(x_{t:t_{c}+\tau_{f}},\hat{g}|d_{t}\right)p\left(z_{t}|d_{t-1}\right)}\left[w\cdot D_{KL}\left[q\left(z_{t}|x_{t:t_{c}+\tau_{f}},\hat{g}\right)∥p\left(z_{t}|d_{t-1}\right)\right]\right]\\ & -E_{p\left(\hat{g}|d_{t}\right)p\left(z_{t}|d_{t-1}\right)}\left[\log\tilde{p}\left(\hat{g}\right)\right])\\ \approx \sum_{t=t_{c}}^{t_{c}+\tau_{f}} & (-E_{p\left(x_{t:t_{c}+\tau_{f}},\hat{g}|d_{t}\right)p\left(z_{t}|d_{t-1}\right)}\left[w\cdot D_{KL}\left[q\left(z_{t}|x_{t:t_{c}+\tau_{f}},\hat{g}\right)∥p(z_{t}|d_{t-1}\right]\right]\\ & +\frac{{\left({\bar{g}}_{t}-\hat{g}\right)}^{2}}{2\sigma^{2}}). \end{array}$$

Here, $\tilde{p}\left(\hat{g}\right)$ denotes the prior-preference distribution, which encodes the preferred goal. The first and second terms correspond to the epistemic and extrinsic terms of the EFE in our proposed framework, respectively. In the original literature [2,17], the expectation in the following equation is taken with respect to the predictive posterior *q*. In our framework, however, the likelihood $p(x_{t:t_{c}+\tau_{f}},\hat{g}|d_{t})$ and the prior $p(z_{t}|d_{t-1})$ fulfill the same role.

Note that the KL divergences in Equations (6) and (7) have opposite signs. This means that during inference—based upon past observations—the agent is trying to minimize the divergence between the approximate and true posterior. Conversely, in the future, the expectation over unobserved outcomes means the agent is trying to maximize the divergence to maximize expected information gain (i.e., epistemic value). We assume the prior preference as a Gaussian with mean equal to the preferred goal, and standard deviation σ. Accordingly, the second term can be calculated as the squared error between the predicted goal ${\bar{g}}_{t}$ and the preferred goal $\hat{g}$, scaled with the variance of prior preference. We model both the likelihood and the prior preference as Gaussian distributions; for the likelihood, the variance is fixed at $\sigma^{2}=1$; for the prior preference, the variance is set to the median absolute deviation $m=\mathrm{median}(|x_{i}^{p}-\mathrm{mean}\left(x_{i}^{p}\right)|)$ computed over the predicted observations $x_{t+1:t+\tau_{f}}^{p}$, where mean(·) denotes the mean of all samples. This implementation is based on the idea that if the deviation among predicted observations (uncertainty) decreases, the possible information gain is small, so the agent should emphasize goal-directed behavior.

**Figure 2:**
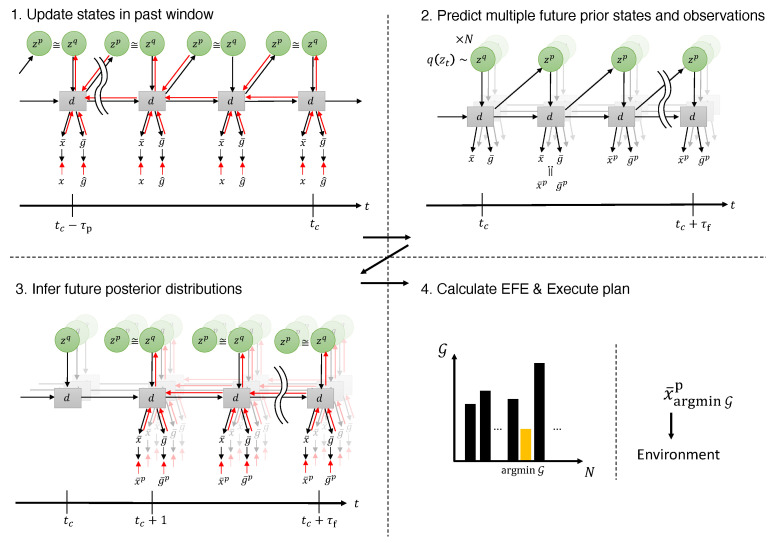
Overview of action plan generation. The planning procedure consists of four major steps. When time step indices are omitted from variables in the figure, the horizontal axis shown at the bottom indicates the corresponding time steps. The overlapped networks in the background represent *N* samples being drawn.

We next describe how the robot generates an action plan by computing free energy in two separate windows. First, for the past window, spanning $\tau_{p}$ steps before the current time step $t_{c}$, we minimize the evidence free energy in Equation (6) online by updating the posterior at each time step in order to fit all the latent variables to the observed sensory sequence (Figure 2, top left).

Second, at time $t_{c}$, we draw *N* samples of the stochastic latent state from the current approximate posterior. Beginning with each sample, we then generate future prior distributions by repeatedly predicting each sampled deterministic latent state forward, predicting the next prior distribution and latent state at every step up to $\tau_{f}$ steps into the future (Figure 2, top right). From every predicted state, we generate future observations $x_{t+1:t+\tau_{f}}^{p}$ and goals $g_{t+1:t+\tau_{f}}^{p}$ by sampling from the corresponding likelihood distributions. Instead of sampling from the likelihood distribution, we follow the variational autoencoder approach [22] and predict its mean directly. Using the predicted observations, we can compute the log marginal likelihood under the prior-preference distribution.

Third, for the future window, we infer approximate posteriors $q\left(z_{t+1:t+\tau_{f}}|x_{t+1:t+\tau_{f}},\hat{g}\right)$ by updating the adaptive vectors so that the evidence free energy is minimized. The KL divergence term of the evidence free energy in the future window can be computed directly from the approximate posterior and the prior distributions. In contrast, the log-likelihood term cannot be evaluated straightforwardly, because the actual future observations and goals are not available in the future window.

In the EFE formulation, the approximate posterior in the future window is conditioned on the predicted observations $x_{t+1:t+\tau_{f}}^{p}$ and predicted goals $g_{t+1:t+\tau_{f}}^{p}$, because the epistemic value is computed under an expectation with respect to the prior over latent states and the likelihood. Accordingly, the log-likelihood term of the evidence free energy in the future window is computed by treating the predicted observations $x_{t+1:t+\tau_{f}}^{p}$ and the predicted goals $g_{t+1:t+\tau_{f}}^{p}$ as the targets of the prediction from the approximate posterior (Figure 2, bottom left).

Finally, having obtained *N* predictive trajectories—each with its prior, approximate posterior, and marginal likelihood under prior preference—we compute the EFE for every trajectory. We then select the proprioceptive observation $x_{t_{c}+1,\arg\min G}^{p}$ at the next step $t_{c}+1$ from the trajectory with the lowest EFE as the action at the next time step (Figure 2, bottom right). Because the EFE comprises the epistemic term that encourages exploration and the extrinsic term that drives goal-directed behavior, this planning procedure supports both exploratory and goal-directed actions. Importantly, the framework can generate actions without relying on explicit policy candidates, a requirement that is common in conventional active-inference approaches. Moreover, the generated action plans remain consistent with past observations because they are produced from the PV-RNN latent state *z*, which encodes information from past observations. Consequently, the framework can exploit this past information to generate action plans that are effective for both environmental exploration and goal achievement.

## 4. Experimental Results

In order to evaluate our proposed model, and to demonstrate its performance, we undertook a series of simulation experiments based on a modified T-maze environment, shown in [3]. Three experiments were considered, from simpler to more complex, so that the logic and findings of the study could be better understood.

In Experiment 1, we first examined the performance of EFE-GLean in a discrete action space, closely following the original experiment in [3]. In this environment, we observed that selecting the policy with the lowest EFE allowed the agent to consistently find the goal, but it also showed how the latent states and resulting policies changed following the EFE.

In Experiment 2, we evaluated the proposed model in a continuous action–sensation domain. In this experiment, the agent was placed in a 2D Cartesian space T-maze. Following the original concept image of the T-maze in Experiment 1, a small area at the end of each of the corridors has colored flooring, and the agent possesses a sensor that can determine the color of the floor underneath it. In this environment, we compared our newly proposed model to our previous model, T-GLean, as well as an agent that acts based on habituation by predicting position and color sensation of the next step while minimizing evidence free energy (habituation-only agent). We observed how maximizing information gain is vital for this task and how the prior-preference variance changed over a long sequence of actions for each agent.

In Experiment 3, we examined how the proposed model can modify its action plan to deal with a dynamically changing world. For this purpose, we extended the T-maze environment to include a randomly placed obstacle at the top edge of the maze which could be sensed by the agent using newly added obstacle sensors. We demonstrated how the action plan can be dynamically changed by adapting the approximate posterior in the past window, as well as in the future window, by simultaneously minimizing the evidence free energy and expected free energy. We also tested the robustness of our model by reducing the past observation window, limiting the contribution of evidence free-energy minimization in plan generation.

The PV-RNN parameters used in the subsequent experiments are noted in Table 1. The values of these parameters were selected empirically based on our previous studies [24]. In particular, it is crucial to allocate a sufficient number of latent units in the model for learning the world model; however, an excessive number of *z* units leads to poor learning due to a large number of independent random variables. These parameters were unchanged between training and testing. The details of how the PV-RNN was trained for each experiment are given in the respective subsections.

### 4.1. Experiment 1

**Figure 3:**
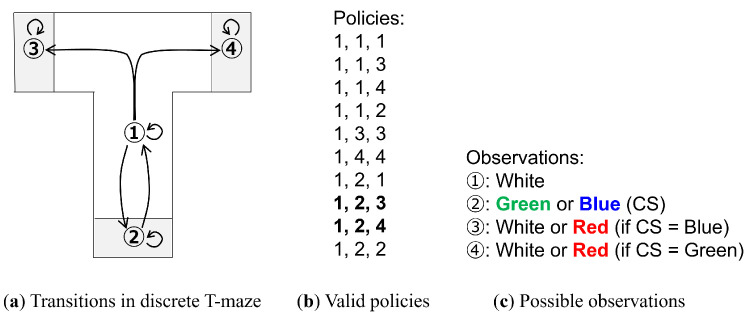
The discrete T-maze simulation. (**a**) The possible transitions between the four states, with 1 corresponding to the state for staying at the center of the T-maze, and 2, 3, and 4 corresponding to the states for the bottom, left, and right ends of each corridor, respectively. The agent always starts at 1, and cannot leave 3 or 4. (**b**) All valid policies following the aforementioned rules, with the preferred policies shown in bold. (**c**) Possible observations at each position. The observation at state 2 is referred to as the conditioning stimulus (CS), as its color indicates the position of the goal (red color, at either state 3 or 4).

Following the experiment in [3], we first undertook an experiment in a discrete action space. As shown in Figure 3a, there are four states that correspond to four possible agent positions in a T-maze: (1) center, (2) bottom, (3) left, and (4) right. This position is encoded as a 4D one-hot vector.

At each of the aforementioned states, a color can be observed. State 1 has a fixed color of white; however, the colors of the remaining states are determined by the color of state 2. State 2 has a 50/50 chance to be either green or blue, and is referred to as the conditioning stimulus (CS). If the CS is blue, then state 3 is red and state 4 is white, and if the CS is green, then the colors of states 3 and 4 are reversed. The observation of the current state is encoded as a 4D one-hot vector of the possible colors: blue, green, red, and white. Note that unlike in [3], the relationship between the CS and the position of the red color is deterministic.

Along with each state having a self-connection, state 1 has transitions to states 2, 3, and 4, while state 2 has a transition to state 1. States 3 and 4 are terminal states. Using policies of length 3, 10 valid policies are enumerated, as shown in Figure 3b.

Finally, an additional 2D one-hot vector, indicating whether the position with the red observation has been reached, is provided as an extrinsic goal. Note that [3] refers to the red color as the unconditioned stimulus (US); however, we refer to it as the goal, following our previous terminology.

The agent always starts at position 1 (center) and has no information on the location of the goal. From an outside observer’s perspective, it is clear that in order to reach the goal reliably, it is desirable to first visit state 2 and check the CS, since it will reveal information on the position of the goal. We note that there are two policies that fit this description (bolded in Figure 3b), and unlike in our previous work, where the human tutor would provide examples of the preferred behavior, the agent must select the appropriate policy from all possible policies. In this context, a preferred policy is one that first checks the CS and then finds the goal.

To train the PV-RNN, 20 training sequences of length 3 were generated, corresponding to all of the valid policies shown in Figure 3b, repeated once for the goal being in either state 2 or 3. The dimensionality of each sequence, as described above, was 10. The PV-RNN was trained for 500,000 epochs using the Adam optimizer and a learning rate of 0.001. The trained PV-RNN was then tested over 100 trials, with each trial having N=100 samples. Each trial was conducted with a different random seed. During testing, inference of both the past approximate posterior and future approximate posterior was performed over 100 iterations per time step, with an increased learning rate of 0.1. After sampling, the policy with the lowest EFE (argmin) was selected. We discuss our policy selection methodology in more detail in Appendix A. In the following results, we examine the agent at the initial time step, after it has observed state 1 and before it moves to the next state.

**Figure 4:**
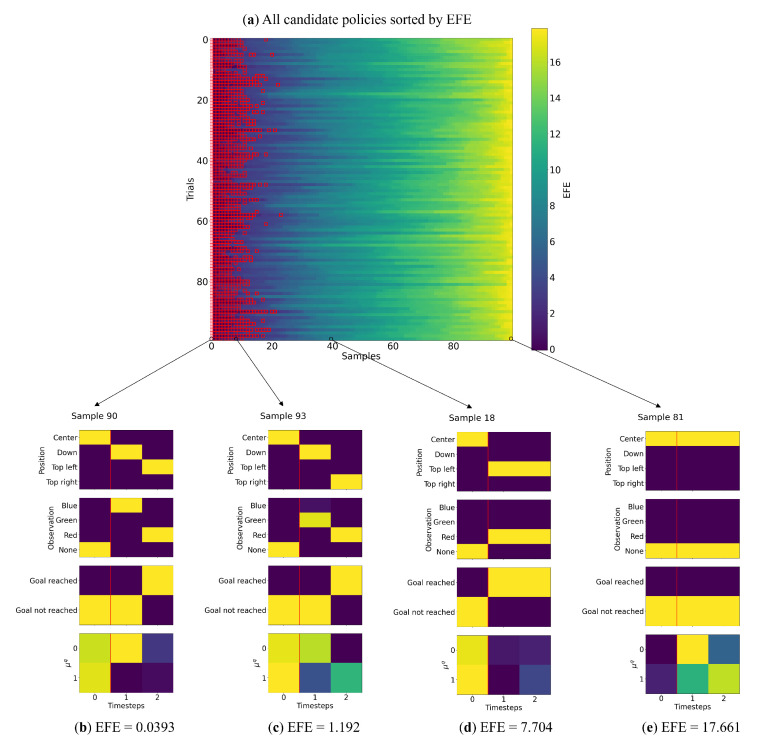
Visual representation of all sampled policies on the discrete T-maze simulation, at the initial time step. (**a**) A heatmap showing all 100 trials (vertical axis) by 100 candidate policies (horizontal axis), sorted by EFE. The policies that match our preferences are highlighted in red. The leftmost column represents the argmin policy that is selected. (**b**–**e**) Analysis of four sample policies from trial 100, at varying levels of EFE. The four subplots represent, from top to bottom, agent position, observation, goal reached, and the $\mu^{q}$ from the stochastic latent states. The red line indicates the first time step has passed, with two time steps in the future.

The results of Experiment 1 are summarized in Table 2. We observe that the PV-RNN-generated policy samples approximating the training distribution—as noted earlier, only 2 of the 10 possible policies follow the preferred pattern, and only one of them will lead to the goal. However, when selecting the lowest-EFE policy, the preferred policy is selected 100% of the time. Moreover, there is a significant difference in the EFE of policies that match our preferences compared to all other policies. We summarize our analysis of this phenomenon in Figure 4.

Figure 4a depicts a graphical representation of the sample selection process when considering all samples. We observed that the preferred policies (highlighted with red boxes) were largely clustered to the left when policies were sorted by lowest EFE. Figure 4b–e break down a single trial, where we observed that the policies with low EFE matched our preferred policies (visiting the CS and then the goal). As the EFE increases, there are policies that have the agent go directly to states 3 or 4, followed by policies that have the agent not move towards the goal at all. From these results we can surmise that the policies likely to be selected, although uncertain about the goal position due to lack of information, will all go to state 2 to maximize information gain.

Observing the latent state activity, there is a difference between policies where the agent visited the CS (Figure 4b,c) compared to those that did not (Figure 4d,e). This may indicate actional intention. Additionally, in this setting, it appears that the cutoff for preferred policies occurs at an EFE value of approximately 2.0, with more diverse policies at higher EFE values.

### 4.2. Experiment 2

Experiment 1 had a limited state space and time horizon; however, our robots operate in a continuous action space and with a much longer time horizon. To simulate this, in this experiment we expanded the discrete T-maze into a 2D T-maze, with the agent position given in 2D Cartesian (x,y) coordinates. The perception of the agent remained, as in the previous experiment, a 4D one-hot vector encoding the four possible colors. The goal-reached vector was replaced with a goal-sensation vector, a 4D one-hot vector encoding the color of the final sensation, which in this experiment could be red or white. This teleological representation of the goal follows our previous goal-directed planning approach in T-GLean. In addition, to be consistent with our previous work, we use the term plan when describing the generated sequences of actions. We note that while a policy, in the context of active inference, contains only future actions, as our focus is on how to select future action sequences using EFE, we consider the terms plan and policy interchangeable.

**Figure 5:**
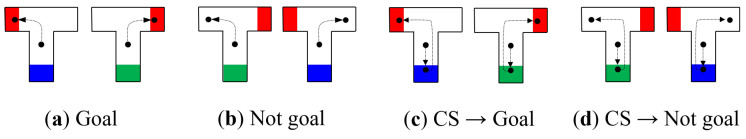
The possible trajectories of the agent in the continuous T-maze. As in the discrete T-maze, the agent starts in the center of the maze. From there it can go directly to one of the possible goal positions (top left or top right), or go down to the conditioning stimulus (CS) before going to a possible goal position. The colored areas represent states 2–4 in the discrete T-maze, with the red area representing the goal and the CS area colored blue or green according to the location of the goal. During training, the agent will experience cases where (**a**) the goal is immediately found, (**b**) the goal is not found, (**c**) the CS is visited and then the goal is subsequently found, and (**d**) the CS is visited and the goal is not found.

As in the discrete T-maze, the agent always starts in the center of the maze, which in this case is at (1.5,1.5). From there, it can follow four possible trajectories, with a 50/50 chance of ending at the goal position. The possible trajectories are summarized in Figure 5. Note that we have removed the cases where the agent remains idle and does not attempt to reach the goal, since as shown in the previous results those plans are very unlikely to be selected by the agent. In this setting, when considered independently, the probability of the agent checking the CS or finding the goal is 0.5.

The agent’s movements were controlled by a simple proportional controller that received (x,y) and translated it to agent position, while sensor noise was simulated as a Gaussian distribution on the position, with σ=0.01 when the agent was in motion and σ=0.005 when stationary. The simulation time interval was set at 0.5. To collect training data, the agent followed a series of waypoints to form trajectories traversing the middle of the corridors, with stops in the center of the colored areas at the ends of the corridors. Due to the aforementioned added noise creating some run-to-run variability, each of the possible trajectories was repeated 10 times, for a total of 80 training trajectories. Each trajectory had a length of 25 time steps and a dimensionality of 10. The PV-RNN was trained for 200,000 epochs, with the optimizer settings being identical to Experiment 1.

In this experiment, we examined the behavior of our agent as it moved through the maze, and compared our current EFE-GLean agent to our previous T-GLean and a “habituation-only” agent described previously. As in Experiment 1, each agent undertook 100 trials with random goal placement, and N=100 samples.

Table 3 summarizes the results of Experiment 2. We note that the EFE-GLean agent was consistently able to find the goal by first checking the CS, as suggested by the previous experiment, while the two baseline approaches, which did not sample EFE, performed significantly worse. The agent applying VFE minimization (habituation-only) showed a CS rate (chance to check the CS) of approximately that of finding it by chance, which follows the training data distribution. The success rate was higher than in the training data, which suggests that the instances where the agent visited the CS had a higher chance of finding the goal.

Our previously proposed T-GLean, which does not consider information gain, did not move back to check the CS at all, instead it always attempted to go directly to the goal, resulting in a random chance of it succeeding in finding the goal. This result is not unexpected, since T-GLean expects a human tutor to demonstrate the appropriate behavior—in this case, the human tutor would manipulate the agent to go down to check the CS, then go to the correct goal position. However, in this setting where the training data does not bias the agent’s behavior, T-GLean’s lack of intrinsic motivation for self-exploration led it to constantly generate suboptimal action plans.

**Figure 6:**
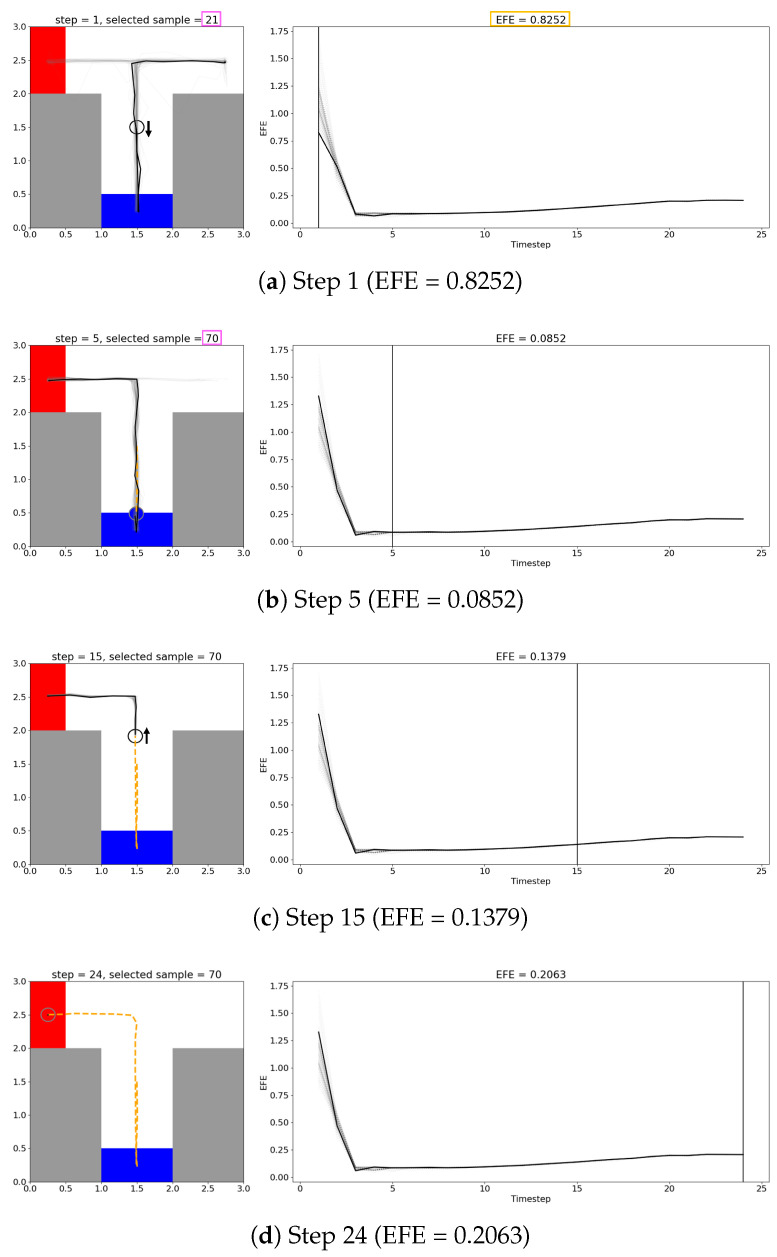
Plan generation during a trial in the continuous T-maze. The sequence (**a**–**d**) shows how the agent’s plans develop as the trial progresses, with the left subplots showing the agent (represented by a circle) in the T-maze, all candidate trajectories (light gray lines), and the selected trajectory (black line), with an arrow indicating the direction the agent will travel. The right subplots show the EFE over time of all candidate plans, with the selected plan’s EFE over time shown as a thick black line. (**a**) Initially, with no information about goal position, there is an even distribution of plans going down, left, and right; selecting the plan with the lowest EFE selects a plan that goes down to the CS. Note that the goal position is still not correct at this point. (**b**) Once the agent receives sensory information at the CS (indicated by the agent changing color), the trajectories all converge on the correct goal position. Note that as the agent approaches the CS, the EFE drops and the agent shifts from curiosity-driven behavior to a stable goal-directed behavior. (**c**,**d**) The agent follows the planned trajectory to the goal, with its past trajectory marked with an orange dashed line.

**Figure 7:**
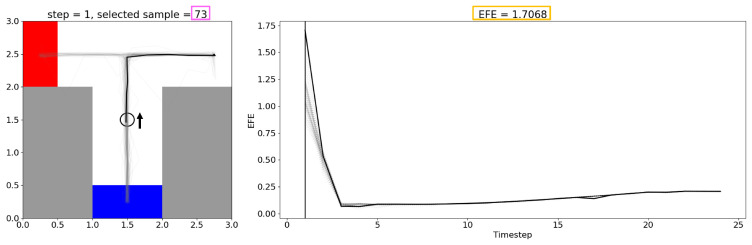
A candidate plan from the same trial as Figure 6 with high EFE (1.7068 compared to 0.8252). This plan has a trajectory that proceeds directly to one of the possible goal areas, which is shorter but suboptimal due to not having any information about the true goal position.

To investigate this, we compared two cases of selecting the lowest- and highest-EFE plans in Figure 6 and Figure 7, respectively. Figure 6 follows the progression of the agent from the start of the trial to the end. A video with several examples from Experiments 2 and 3 is available at https://youtu.be/I_R2tFh_OxY (accessed on 19 June 2025). The thick black line in the left subplots indicates the selected plan’s trajectory, while the thick black line in the right subplots shows the selected plan’s EFE at each step over the course of the trial. Note that all other candidate plans’ trajectories and EFEs are overlaid in a light gray for reference. At the initial step, where the agent starts with no information, there was a diverse set of candidate plans. The plan with the lowest EFE was selected, which was a plan that moves the agent back to check the CS. During this phase, where the future uncertainty is high (as seen by a diverse set of trajectories the agent could follow), the agent tends to prefer exploratory behavior, as shown by the preference towards plans that move the agent to the CS.

As the agent moves and updates its possible future observations and actions, the ceiling on future information gain naturally declines, corresponding to the decreasing EFE, that reached a minimum as the agent approached the CS. This behavior is in line with the expectation that checking the CS grants the agent all the necessary information to reach the goal, and that further exploration is unnecessary. Once the agent observes the CS, the agent preferentially exhibits exploitative goal-seeking behavior, where all candidate plans are optimized to reach the now-known goal position. We examine the activities of latent variables during the exploration and exploitation periods in a comparative manner in Appendix B.

Figure 7 shows the plan with the highest EFE, plotted in the same way as Figure 6a. While this plan takes the shortest path directly to a possible goal, such plans tend to have a high EFE and are not preferred by EFE-GLean.

We note that in both Figure 6 and Figure 7, as the trial continues and the agent moves towards the goal, the EFE appears to slowly rise, which could switch the agent back to exploratory behavior. However, this “bored” behavior is not desirable for our goal-directed agent, as this can cause destabilization of the PV-RNN internal states and potentially unsafe movements of the agent.

**Figure 8:**
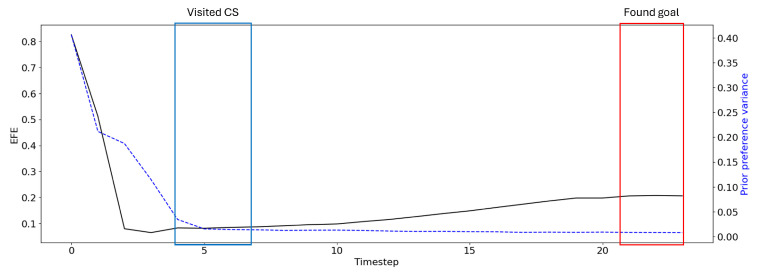
A plot showing the EFE for the plan in Figure 6a. Here, EFE (black solid line) is shown without considering prior-preference variance, which results in the EFE increasing as the trial continues. The prior-preference variance (blue dashed line), which is computed as the median absolute deviation over all candidate plans, is a measure of future uncertainty, with low uncertainty favoring extrinsic value maximization. The uncertainty is highest at the beginning, where the agent has no information on the goal position and has the most possible actions.

As such, as described in Section 3, we computed the prior-preference variance in G in order to manage the preference between maximizing epistemic and extrinsic values. To analyze the relationship between prior-preference variance and EFE, we present EFE without prior-preference variance (i.e., $\sigma^{2}=1.0$). Figure 8 compares the EFE of a given plan without considering prior preference variance to the calculated prior-preference variance. In the condition where the agent is certain of its future, the prior preference is strong and thus the agent prefers to maximize extrinsic value, resulting in stable goal-directed behavior over an extended time horizon.

The results of this experiment in a longer continuous action space demonstrated that our agent was consistently able to exploit information gain maximization until the goal position became certain, at which point stable goal-directed behavior was exhibited with reduction of the prior-preference variance.

Equivalently, when the goal position became certain, there was an increase in the precision of the prior preferences. This is similar to the simulations of dopamine discharges based upon the precision of posteriors over policies explored in prior work [25]. Here, we are using a simpler setup and the adaptive precision has been specified heuristically (as opposed to being optimized with respect to variational free energy). However, the similarity in the dynamics of the implicit precision is interesting.

### 4.3. Experiment 3

Our previous study demonstrated that the T-GLean agent can adjust its plan dynamically when the environment changes, and the current study evaluated this capability of EFE-GLean. This was accomplished through optimization of the adaptive vector (A), as demonstrated in Appendix C. Experiment 3 tested whether the EFE-GLean agent could prepare action plans in an extended T-maze task when anticipating an obstacle hidden in the environment, and could update the plans adequately when the obstacle was actually sensed.

Following on from Experiment 2, the T-maze environment was extended by adding a randomly placed obstacle along the top wall, outside of the goal areas. The obstacle blocked half of the corridor, requiring the agent to take a detour to avoid collision. In order to detect the obstacle, the agent was equipped with four range sensors placed at 45 degrees to the cardinal directions. These four sensors return a floating point value between 0.0 and 0.7, sufficient to sense its immediate surroundings (the corridor width is 1.0). The obstacle is always outside of the range of the sensors at the initial position of the agent. In addition, the color representation was changed from a one-hot vector to a more realistic RGB representation, with each channel being a floating point value between 0.0 and 1.0. The colors in the environment remained unchanged. The agent position remained represented by a Cartesian (x,y) coordinate; however, the simulator time interval was reduced to 0.2. In this way, the agent was given additional time steps to sense the obstacle, slow down, and maneuver around it if necessary.

The training data was collected in a similar fashion to Experiment 2, except now with each of the eight possible patterns shown in Figure 5—four training sequences had a randomly placed obstacle along the top edge of the maze and another four sequences had no obstacle—for a total of 64 training sequences. While collecting data, the agent attempts to remain centered in the corridor by maintaining an equal distance based on observations from its range sensors, and slows down when taking the narrow path next to the obstacle. The total number of time steps was extended to 60, with each sequence having a dimensionality of 12. The PV-RNN was otherwise configured and trained as in Experiment 2.

To analyze the robustness of EFE-GLean, we conducted an ablation study where we reduced the past window $\tau_{p}$, which reduces the evidence free energy, as given in Equation (6). The results are summarized in Table 4. When we reduced $\tau_{p}$ to half length (30 steps), we observed that while the exploration behavior remained intact, the goal-directed behavior became degraded, as the agent appeared to forget the CS in some cases and select a plan to go to the incorrect goal position.

Put simply, the minimization of variational free energy provides posterior beliefs about the current states of the world. These beliefs are then used to evaluate the expected free energy using posterior predictive densities in the future. This means if the agent is uncertain about the current state of affairs—due to a reduction in $\tau_{p}$—it will also be uncertain about the future and the consequences of the different policies it could pursue.

In the extreme case where $\tau_{p}$ was limited to a single step, the success rate degraded to below the training baseline, largely due to the agent being no longer able to adequately navigate around obstacles.

**Figure 9:**
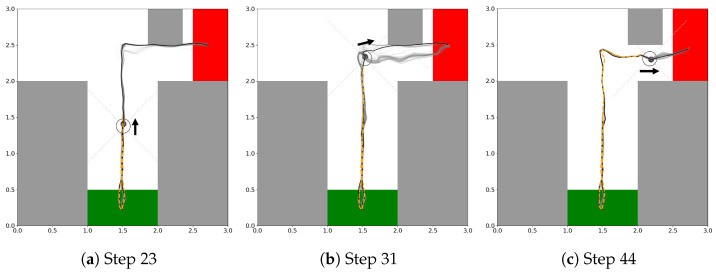
Development of the plan trajectory generated by the original EFE-GLean agent in a trial in the extended continuous T-maze with an obstacle placed in it. The thick black trajectory represents the selected plan with the minimum EFE. The obstacle (gray square) can appear with a uniform distribution along the top wall of the maze. (**a**) after checking the CS at time step 23, (**b**) immediately after detecting the obstacle at time step 31, and (**c**) when passing through the obstacle at time step 44.

In the analysis of the plan trajectories dynamically generated during travels of the original EFE-GLean agent, some interesting behavioral properties were observed (a typical example is shown in Figure 9). In Figure 9a, after checking the CS, the agent generated the shortest path going toward the goal which could collide with the obstacle since it had not yet been detected. However, there was some uncertainty in the trajectories due to the possibility of the obstacle being located at various positions. In Figure 9b, immediately after detecting the obstacle, two plan options, one for taking the shortest path to the goal and the other for detouring around the obstacle, were generated before the convergence of the plan. Finally, in Figure 9c, the plan converged to the one that stably arrives at the goal by detouring around the obstacle.

These experimental results demonstrate that EFE-GLean retains the ability to rapidly adjust its plan independently of its exploration behavior. As shown in our previous work, this is an important ability when operating robots in the physical world with the potential for sudden environmental changes.

## 5. Discussion

This study proposed and validated EFE-GLean, a novel extension of the T-GLean model, which integrates epistemic value into low-dimensional active inference planning. Our results demonstrate that incorporating expected information gain enables agents to effectively balance exploration and exploitation within continuous action domains. Through a series of experiments in both discrete and continuous T-maze environments—including settings with dynamically changing obstacles—we showed that EFE-GLean not only achieves goal-directed behavior with high success rates, but also adapts in real time to unexpected environmental changes. This performance is driven by the model’s ability to simultaneously minimize past variational free energy and future expected free energy, facilitating dynamic plan revision based on hidden state exploration. These findings highlight the utility of embedding curiosity-driven mechanisms into compact latent planning architectures, supporting robust and flexible behavior in partially observable and dynamically evolving environments.

A key future direction is to apply EFE-GLean to humanoid robots, such as iCub, with high-dimensional motor spaces. Prior work [15] has demonstrated an active inference framework for selecting between goal-directed and exploratory behavior on a Rakuda-2 robot, and other works have demonstrated the feasibility of active inference under noisy, uncertain real-world conditions, performing adaptive reaching and head tracking via predictive body perception models [26,27]. We hypothesize that by constraining the policy search to a low-dimensional latent space, our method can scale to such complex embodiments without exhaustive exploration of the full motor command space. Future experiments with physical humanoids will help verify the scalability and adaptability of EFE-GLean in real-world, high-dimensional action spaces.

While our current implementation uses random sampling in the latent space to select future trajectories, this approach can be computationally costly and suboptimal in scenarios requiring rapid replanning. An alternative strategy is to apply gradient descent in the latent space, which could enable faster convergence and more sample-efficient optimization. Previous research in active inference controllers has shown that optimization-based approaches can yield robust real-time performance, particularly when using continuous approximations of variational free energy [28]. Incorporating such gradient-based techniques may improve the responsiveness of EFE-GLean, especially for continuous control tasks.

As our framework leverages PV-RNN, we have relied on conventional BPTT for learning. However, other biologically plausible approaches that use only local information such as eligibility traces [29,30] and predictive-coding schemes that converge to BPTT solutions [31] offer interesting alternatives to BPTT that could be incorporated into our framework in future work.

Another rich avenue for future exploration is combining EFE-GLean with mechanisms for incremental or developmental learning. Robots that accumulate structured experience over time—adjusting their inference and planning strategies as they mature—may more closely mimic human learning patterns. Architectures like SAGG-RIAC have shown how intrinsic motivation and goal exploration can facilitate efficient learning in high-dimensional sensorimotor spaces [32]. Extending EFE-GLean with such adaptive learning dynamics could support long-term skill development and autonomous behavior refinement.

## Figures and Tables

**Table 1:** PV-RNN parameters used for all experiments. $R^{d}$ and $R^{z}$ refer to the number of deterministic (*d*) units and probabilistic (*z*) units, respectively. τ is the time constant of the layer, while *w* refers to the meta-prior, excluding the initial time step, where it is fixed to 1.0.

(a) Experiment 1				
Layer *l*	$R^{d}$	$R^{z}$	τ	*w*
1	10	2	1	0.1
(b) Experiment 2 & 3				
Layer *l*	$R^{d}$	$R^{z}$	τ	*w*
1	80	6	2	0.005
2	20	3	8	0.01

**Table 2:** Results for Experiment 1. “Preferred policy selected” refers to the percentage of trials (n=100) where one of the preferred policies was selected. “Preferred policy samples” refers to the percentage of all samples (n×N = 10,000) generated that matched one of our preferred policies. “Avg preferred policy EFE” and “Avg other policy EFE” refer to the mean and standard deviation of the computed expected free energy for the preferred policies and the other policies, respectively.

Preferred Policy Selected	Preferred Policy Samples	Avg Preferred Policy EFE	Avg Other Policy EFE
100%	10.09%	0.747±0.744	10.15±3.82

**Table 3:** Results for Experiment 2. The algorithms under test are the proposed EFE-GLean, a habituation-only agent, and our previous T-GLean model. CS rate refers to the proportion of trials in which the agent checked the CS at the bottom of the maze. Success rate is the proportion of trials where the agent successfully found the red goal. The best result is highlighted in bold. The number of trials is n=100.

Algorithm	CS Rate	Success Rate
**EFE-GLean**	96%	94%
Habituation-only	55%	70%
T-GLean	0%	60%

**Table 4:** Results for Experiment 3, an ablation study on the past window length. CS rate refers to the proportion of trials in which the agent checked the CS at the bottom of the maze. Success rate is the proportion of trials where the agent successfully found the red goal. The number of trials is n=100.

Algorithm	CS Rate	Success Rate
**Original EFE-GLean** ($\tau_{p}=60$)	96%	97%
EFE-GLean ($\tau_{p}=30$)	96%	74%
EFE-GLean ($\tau_{p}=1$)	100%	36%

**Figure A1:**
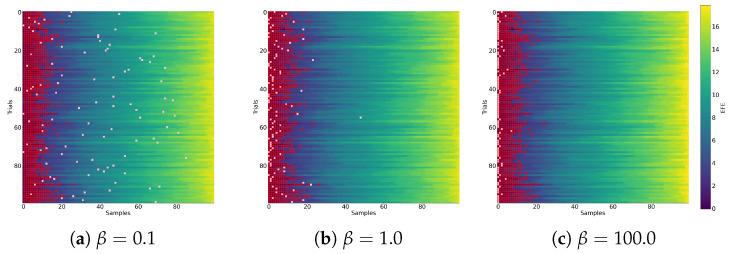
A visual representation of the selected policies. Each row shows the sorted G; the red highlighted blocks are preferred policies and the pink filled blocks are the selected policies.

**Figure A2:**
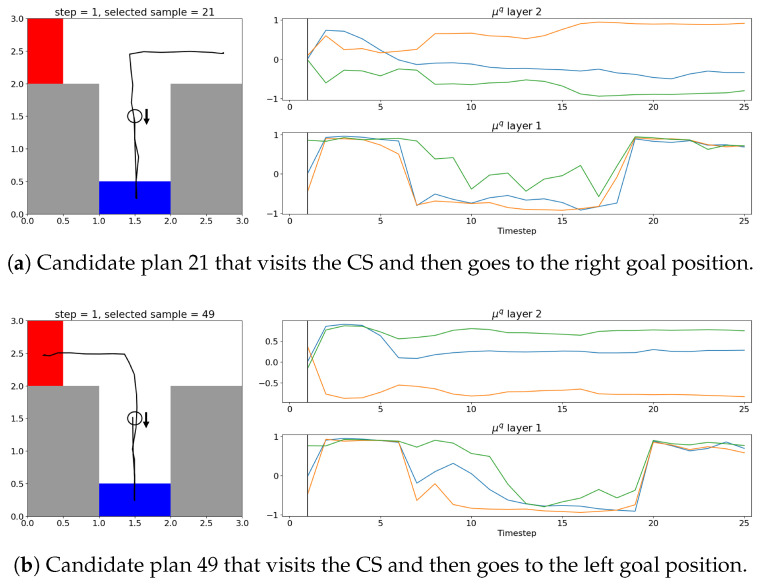
Two candidate plans generated at t=1 that end in different goal positions. Note that the agent has no information on the true goal position at this time step.

**Figure A3:**
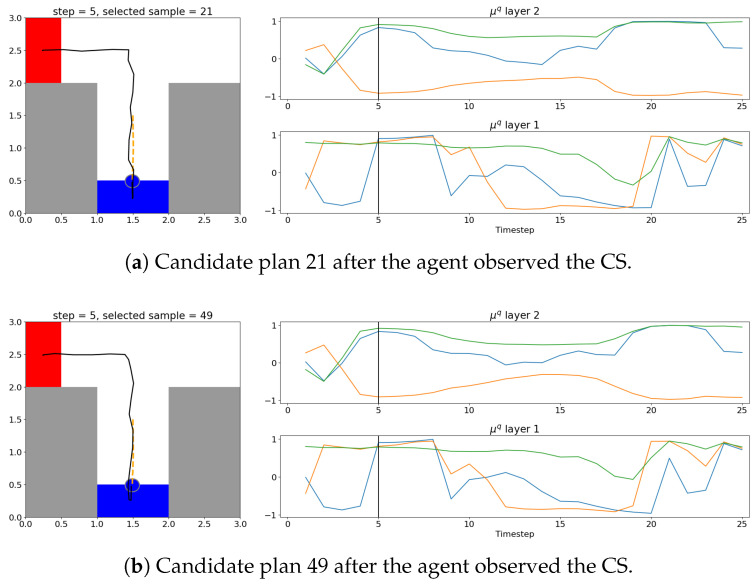
The two candidate plans from Figure A2 now at t=5, after the agent observes the CS.

**Figure A4:**
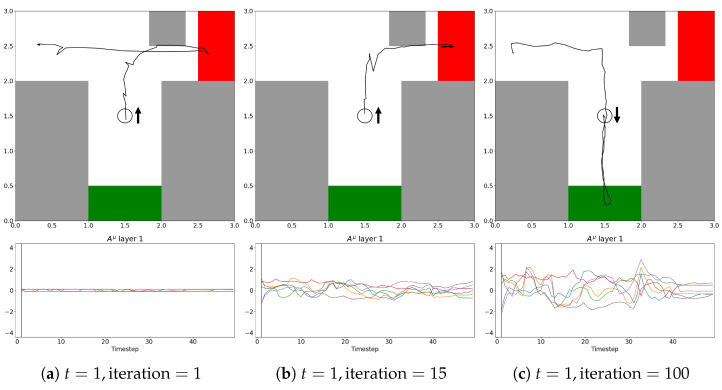
Top: Plots of the agent in the T-maze, with the black line representing the planned trajectory. The agent cannot sense the obstacle in the top right from its current position. Bottom: Plot of $A^{\mu }$, with the *X* axis representing time steps and the *Y* axis the value of each of the six A variables. (**a**) From the initial value $A_{t=0}=0$, the generated action plan is far from ideal. The plot of A shows a small update has occurred after one iteration. (**b**) After 15 optimization iterations, the A variables have shifted and are now generating a more plausible action plan. (**c**) At the final iteration, the generated action plan looks close to optimal (note that the agent does not know where the goal is at this time step).

**Figure A5:**
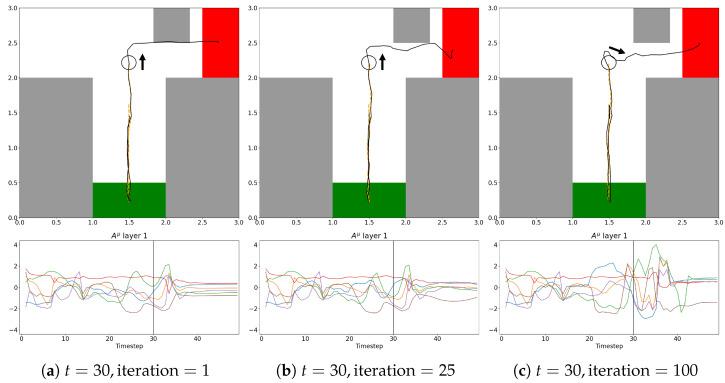
The agent at t=30 can now sense the obstacle ahead. (**a**) Initially, the planned trajectory would have the agent collide with the object. Note that the A variables have only updated slightly since the initial plan at t=1, as expected in a static environment. (**b**) After 25 optimization iterations, the A variables have shifted so the agent can try to take a path that avoids the obstacle; however, this does not appear to be sufficient at this point. (**c**) At the final iteration, the A variables have shifted considerably and the agent now plans to detour around the obstacle.

## Data Availability

The code and datasets used for the experiments in this study are available at https://github.com/oist-cnru/EFE-GLean (accessed on 19 June 2025).

## Appendix A

In this study we used the following deterministic rule (argmin) for policy selection, where $\pi^{*}$ is the selected policy.

(A1) $$\pi^{*}=\arg\min_{\pi }G\left(\pi \right)$$

It is typical for the expected free-energy values G to be converted into a probability distribution over all policies using the softmax function [6,33], from which a policy can be sampled as follows, where β=1/τ, the inverse of the softmax temperature.

(A2) $$P_{\beta }\left(\pi \right)=\frac{\exp(-\beta G(\pi ))}{\sum_{\pi^{\prime }}\exp\left(-\beta G\left(\pi^{\prime }\right)\right)}$$

Based on the aforementioned formulation, the probability of the deterministic and stochastic sampling diverging can be given as follows, where $\Delta_{i}$ is the difference between the minimum EFE and the EFE of the *i*-th policy $G_{i}-G_{1}$, given G is sorted in ascending order and i>1.

(A3) $$\begin{array}{cc} P(\pi_{\beta }^{*}\neq \pi^{*}) & =1-\frac{1}{1+\sum_{i}\exp\left(-\beta \Delta_{i}\right)}\leq \sum_{i}e^{-\beta \Delta_{i}}\leq \left(N-1\right)e^{-\beta \Delta_{min}},\\ \Delta_{min} & =G_{2}-G_{1}. \end{array}$$

Rearranging for β, it is apparent that for any given probability s of selecting argmin G, there is a minimum value of β that will result in the given probability value.

(A4) $$\beta \geq \frac{1}{\Delta_{min}}\log\frac{N-1}{1-s}$$

It follows that as β→∞, the distribution collapses to the argmin value. This is observed empirically in Table A1, which extends Experiment 1 with softmax sampling of G conducted with different values of β. Three examples are also shown in Figure A1.

**Table A1:** Comparison of different values of β when sampling from G. $P(\pi_{\beta }^{*}=\pi^{*})$ is the probability of selecting the same policy as argmin G, while $P(\pi_{\beta }^{*}=\pi_{\mathrm{preferred}})$ is the probability the selected policy is one of the preferred policies.

β	$P(\pi_{\beta }^{*}=\pi^{*})$	$P(\pi_{\beta }^{*}=\pi_{\mathrm{preferred}})$
0.1	0.03	0.21
1.0	0.18	0.83
10.0	0.43	1.0
100.0	0.74	1.0
*argmin*	*1.0*	*1.0*

We observed that for a sufficiently high value of β, the behavior of sampling and argmin G converged. Since the epistemic term of G already maximizes information gain, sufficient exploration behavior occurs without additional sampling. Thus, argmin G is a stable and computationally efficient approximation of softmax sampling in this case.

## Appendix B

While in Experiment 2 we examined the behavior of the agent as it shifted from an exploration preference to an exploitation preference, here we examined the random latent variables of the PV-RNN, focusing on $\mu^{q}$ of the posterior distributions (six in layer 1 and three in layer 2). Note that for clarity, we have selected three random latent variables in layer 1 that showed strong activity.

Figure A2 shows the agent at the starting position at t=1, where the exploration phase begins. Here, several types of future actions are planned: Figure A2a shows a candidate plan where the agent goes right after visiting the CS, and Figure A2b shows another candidate plan where the agent goes left after visiting the CS. We observed that the activity of $\mu_{q}$ between these two plans is quite different.

After the agent observes the CS, it enters an exploitation phase, where all future plan trajectories converge to a single trajectory that reaches the true goal position as shown in Figure A3a,b. It can be seen that in this phase $\mu^{q}$ in both layers is very similar between these two plans. It is also noted that $\mu^{q}$ in the past window changes significantly once the CS is perceived, suggesting an update of past beliefs.

## Appendix C

Optimization of the approximate posterior latent variables (adaptive vector A) is the primary method by which our model generates an action plan, and also how plans are adjusted based on the error between predicted and actual observations. Here, we investigated this aspect by examining how A was updated during Experiment 3.

As described in Section 3 and in our previous work, during inference the PV-RNN weight matrices are fixed and the A variables are optimized using the evidence free energy (Equation (6)) and expected free energy (Equation (7)) as a loss function. The A variables are split into $A^{\mu }$ and $A^{\sigma }$, which map to the approximate posterior $z^{q}$ as described in Equations (3) and (5); however, for clarity of explanation we will focus only on $A^{\mu }$.

The A variables are optimized for 100 iterations for every action the agent takes, with A initialized as $A_{t=0}=0$. As shown in Figure A4a, the initial generated action plan is far from optimal, and is in fact not a trajectory that has been trained. However, as the A variables are optimized, a more feasible action plan is generated. Note that at the initial step t=1, the agent has no useful information other than the preferred goal $\hat{g}$. Thus, at this point optimization of A occurs by minimization of expected free energy.

When the agent senses the obstacle in its path, as shown in Figure A5, after a number of optimization iterations the planned trajectory begins to shift to avoid collision with the obstacle. Note that, as seen in Figure A5c, the optimization makes significant changes in the past A variables, which suggests that the agent is updating its past beliefs regarding the presence of the obstacle.
